# The role of ADHD genetic risk in mid-to-late life somatic health conditions

**DOI:** 10.1038/s41398-022-01919-9

**Published:** 2022-04-11

**Authors:** Miguel Garcia-Argibay, Ebba du Rietz, Yi Lu, Joanna Martin, Elis Haan, Kelli Lehto, Sarah E. Bergen, Paul Lichtenstein, Henrik Larsson, Isabell Brikell

**Affiliations:** 1 School of Medical Sciences, Örebro University, Örebro, Sweden; 2 Department of Medical Epidemiology and Biostatistics, Karolinska Institutet, Stockholm, Sweden; 3 MRC Centre for Neuropsychiatric Genetics and Genomics, Cardiff University, Cardiff, UK; 4 Estonian Genome Centre, Institute of Genomics, University of Tartu, Tartu, Estonia

**Keywords:** ADHD, Clinical genetics

## Abstract

Growing evidence suggests that ADHD, an early onset neurodevelopmental disorder, is associated with poor somatic health in adulthood. However, the mechanisms underlying these associations are poorly understood. Here, we tested whether ADHD polygenic risk scores (PRS) are associated with mid-to-late life somatic health in a general population sample. Furthermore, we explored whether potential associations were moderated and mediated by life-course risk factors. We derived ADHD-PRS in 10,645 Swedish twins born between 1911 and 1958. Sixteen cardiometabolic, autoimmune/inflammatory, and neurological health conditions were evaluated using self-report (age range at measure 42–88 years) and clinical diagnoses defined by International Classification of Diseases codes in national registers. We estimated associations of ADHD-PRS with somatic outcomes using generalized estimating equations, and tested moderation and mediation of these associations by four life-course risk factors (education level, body mass index [BMI], tobacco use, alcohol misuse). Results showed that higher ADHD-PRS were associated with increased risk of seven somatic outcomes (heart failure, cerebro- and peripheral vascular disease, obesity, type 1 diabetes, rheumatoid arthritis, and migraine) with odds ratios ranging 1.07 to 1.20. We observed significant mediation effects by education, BMI, tobacco use, and alcohol misuse, primarily for associations of ADHD-PRS with cardiometabolic outcomes. No moderation effects survived multiple testing correction. Our findings suggests that higher ADHD genetic liability confers a modest risk increase for several somatic health problems in mid-to-late life, particularly in the cardiometabolic domain. These associations were observable in the general population, even in the absence of medical treatment for ADHD, and appear to be in part mediated by life-course risk factors.

## Introduction

Attention-deficit/hyperactivity disorder (ADHD) is an early onset neurodevelopmental disorder with an estimated prevalence of 5–7% in childhood [1]. There is growing evidence that ADHD often persists, with around 15% of affected children continuing to meet full diagnostic criteria by early adulthood and an additional 50–70% experiencing impairing symptoms [2, 3]. In the last decade, prevalence and treatment of clinical ADHD has increased both in children and adults, likely due to increased awareness and access to services [1, 4]. Around 2–5% of the adult population are estimated to have ADHD, yet it remains underdiagnosed in adults [5] and there is limited data on how ADHD impacts health across the lifespan.

Two recent reviews concluded that ADHD is robustly associated with obesity, sleep disorders, epilepsy, migraine, and asthma. Further, increasing evidence supports associations of ADHD with cardiovascular disease, neurodegenerative disorders, and certain autoimmune/inflammatory conditions [6, 7]. Some of these association may be genetically mediated, as small-to-moderate genetic correlations based on genome-wide association studies (GWAS) have been reported between ADHD and body mass index (BMI), obesity, rheumatoid arthritis, psoriasis, and migraine [8–10]. Such genetic correlations may reflect shared biology, whereby the same genetic variants that increase the risk for ADHD also directly increase risk for somatic conditions. However, the effect of ADHD genetic liability on somatic health may also be mediated or moderated by behavioral (e.g., tobacco use and alcohol misuse), cardiometabolic (e.g., high BMI) and socioeconomic (e.g., lower educational attainment) risk factors associated with ADHD [11]. Few studies have tested such hypotheses, likely due to the scarcity of samples in mid-to-late life populations with assessments of both ADHD, somatic health and genomic data. Another challenge is to disentangle the effect of ADHD itself from the effect of ADHD medication treatment. This is an important issue given that ADHD medication rates are high in clinical populations [12–14] and it has been suggested that ADHD medications may confer increased risk of various somatic health outcomes. For example, minor increases in blood pressure are a common side-effect of ADHD stimulant medications, leading to concerns that stimulants may increase risk of cardiovascular disease, although evidence for this is limited [15]. Conversely, ADHD medication treatment may also reduce the risk of negative health behaviors, such as substance misuse [16], and could thereby be hypothesized to lower the risk of certain poor somatic health outcomes.

One approach to partly circumvent these limitations is to study the effect of ADHD genetic liability in population-based samples that are untreated for ADHD, using polygenic risk scores (PRS), which capture the sum of an individual’s common autosomal genetic variant liability for ADHD based on independent genetic discovery data [17]. Such approach can be meaningful given that considerable evidence from family, twin, and molecular genetic studies support that ADHD (1) is heritable, (2) exists as dimensional traits in the population, and (3) such traits are underpinned by a similar genetic architecture as the clinical manifestation of the disorder [18–20]. Indeed, a recent meta-analyses [20] reported that the ADHD-PRS were associated not only with diagnosed ADHD, but also with ADHD traits in the general population across all 26 reviewed studies [20]. This suggests that ADHD-PRS can be used to study the effect of ADHD genetic liability on associated health outcomes, even in samples where ADHD symptoms and diagnosis have not been assessed. Two prior studies have used this approach in the UK Biobank, a large, predominantly middle-aged “healthy and wealthy” population sample, including few (<100) clinically treated ADHD cases. Both studies reported significant association of ADHD-PRS with higher BMI, as well as sociodemographic and life-course risk factors, including lower educational attainment, smoking and alcohol consumption [21, 22]. Thus, it can be hypothesized that higher genetic liability for ADHD is associated with worse somatic health in later life, and that such associations may in part be mediated/moderated by life-course risk factors. However, several somatic conditions have not been assessed in prior studies, and mediation or moderation by life-course risk factors linked to ADHD and ADHD genetic liability remain to be explored [9, 11, 21–24]. The aims of the current study were therefore to (1) test whether genetic liability for ADHD, as captured by ADHD-PRS, is associated with mid-to-late somatic health conditions in a population sample where few are treated for ADHD, (2) explore whether these potential associations are moderated or mediated by life-course risk factors previously associated with ADHD.

## Methods

### Study population

All twins born in Sweden in 1958 or earlier (*N* = 52,080) were contacted for a telephone interview between 1998 and 2002 in the Screening Across the Lifespan Twin (SALT) study. In total, 44,919 individuals completed the SALT interview (response rate 85%). Study design and data collection has been described in detail elsewhere [25–27]. Between 2004 and 2008, SALT participants were re-contacted and asked to donate blood in the TwinGene study; 12,614 individuals donated blood (response rate 56%) and all dizygotic twins and one twin from each monozygotic twin pair (*n* = 9896) were genotyped using the Illumina OmniExpress BeadChip (700 K). Non-genotyped monozygotic twins were imputed from their genotyped co-twin. Details are provided elsewhere [28]. Genetic data was imputed against the 1000 Genomes Project phase 1 version 3 panel. Information on quality control and imputation are provided in the supplement. The final analytic sample size for this study was 10,645 individuals with both genotype and phenotype data.

### Polygenic risk scores

ADHD-PRS were derived in the SALT target sample as the sum of the SNP dosages weighted by the allelic effect from the discovery set across all single nucleotide polymorphisms (SNP) under eight different *p*-value thresholds (0.001 ≤ *P*_*T*_ ≤ 1). SNP weights were obtained from summary statistics of the largest available ADHD GWAS meta-analysis (19,099 cases, 34,194 controls) [9], restricted to European ancestry. The discovery data and target sample were independent. Indels, multi-allelic and symmetric/ambiguous SNPs were excluded, and SNPs were further filtered on imputation quality (INFO < 0.8). To select a relatively independent set of SNPs for the PRS calculation, we ran linkage disequilibrium (LD) clumping (*r*^2^ < 0.1 in 1 Mb window) on the overlapping SNPs in the target and discovery data, using the 1000 Genomes Project European samples as the LD reference. We calculated PRS for each individual in the target sample as the sum of the SNP dosages weighted by the effect from the discovery set across all SNPs under eight specified *p*-value thresholds (≤0.001, ≤0.01, ≤0.05, ≤0.1, ≤0.2, ≤0.3, ≤0.5, and ≤1) using PLINK v1.9 (commands –score –q-score-range) [29]. All *ß*-values were recoded to be positive. We then performed a principal component analysis of all PRS (standardized to a mean=0 and a standard deviation [SD] = 1) across the *p-*value thresholds, retaining the first PC to derive a single (PC-)PRS used in subsequent analyses. This approach has been shown to maximize the amount of variance captured across PRS *p*-thresholds, whilst avoiding overfitting and maintaining a correct Type I error rate [30].

To account for potential population stratification, we derived population covariates (PCs) using principal components analysis implemented in PLINK.v.1.9 [29]. Allele frequencies were obtained for a set of LD-pruned SNPs in unrelated individuals. PCs were estimated by calculating variant weights on unrelated individuals and then projecting remaining samples to the PC scales set by these unrelated individuals. The first 6 PCs were used as covariates in subsequent analyses.

### Somatic health outcomes

SALT participants answered a checklist of whether they had ever been diagnosed or received medical treatment for a wide range of somatic health conditions. Details of the checklist, its use for disease screening, and specificity and sensitivity for each condition, are reported elsewhere [25]. We selected 16 cardiometabolic, autoimmune/inflammatory, and neurological conditions previously associated with ADHD (Table 1) [7]. We focused on conditions with peak prevalence in mid-to-late life, as well as type 1 diabetes and epilepsy, which may onset in childhood but can be chronic, or onset in later life as the consequence of other health problems. We also included obesity given the replicated strong link to ADHD [31], and because our obesity indicators were clinical outpatient diagnoses from 2001 onwards, and/or self-reported largest ever weight, and thus very likely reflect obesity in adulthood. We obtained clinical diagnoses of the same conditions from the Swedish National Patient Register (NPR), and further included register information on sleep disorders and dementia, which were not available via self-report. The NPR includes inpatient care since 1964 and specialist outpatient care since 2001 coded according the International Classification of Diseases (ICD-8 1968–1986; ICD-9 1987–1996; ICD-10 1997-onward) [32]. We identified additional cases using the Swedish Cause of Death Register, with near complete coverage from 1952 [33]. For migraine, sleep disorders, dementia, and Parkinson’s disease, we also identified cases using dispensations of disorder-specific drugs from the Prescribed Drug Register, which records all dispensed prescriptions in Sweden since 2005 coded according to the Anatomical Therapeutic Chemical (ATC) classification system [34]. ICD and ATC codes used to define register-based outcomes are presented in Supplementary Table S1. All outcomes were treated as lifetime (0/1) and defined by either a register-based ICD diagnosis, drug dispensation, and/or self-report.

**Table 1:** *N* cases and % prevalence of lifetime evaluated somatic health outcomes (*N* = 10,645).

Disease area/outcome	Combined	Register-based	Self-reported
*Cardio-metabolic*			
Ischemic heart disease	1206 (11.3)	1104 (10.4)	231 (2.2)
Heart failure	1174 (11.0)	453 (4.3)	843 (7.9)
Cerebrovascular disease	1686 (15.8)	1431 (13.4)	760 (7.1)
Peripheral vascular disease	523 (4.9)	408 (3.8)	157 (1.5)
Hypertension	2730 (25.6)	735 (6.9)	2379 (22.4)
Obesity	1538 (14.4)	67 (0.6)	1526 (14.6)
Type 2 Diabetes	554 (5.2)	385 (3.6)	347 (3.3)
*Autoimmune/inflammatory disease*			
Type 1 Diabetes	163 (1.5)	161 (1.5)	46 (0.4)
Rheumatoid arthritis	594 (5.6)	163 (1.5)	521 (4.9)
Psoriasis	605 (5.7)	195 (1.8)	503 (4.7)
Inflammatory bowel disease	243 (2.3)	176 (1.7)	153 (1.4)
*Neurology*			
Migraine	2207 (20.7)	298 (2.8)	2125 (20.0)
Epilepsy	231 (2.2)	116 (1.1)	121 (1.2)
Dementia	374 (3.5)^a^	374 (3.5)	N/A
Parkinson disease and parkinsonism	473 (4.4)	472 (4.4)	10 (0.1)
Sleep disorder	1272 (11.9)^a^	1272 (11.9)	N/A

Register-based outcomes were treated as lifetime and defined based on a discharge diagnosis in the National Patient Register, the Cause of death register or disorder-specific drug dispensations. Self-report refers to questions answered as part of the SALT interview and were phrased as lifetime. Combined prevalence refers to the total number of cases identified via National Registers, self-report, or both. For details, see methods and Table S1.

^a^Prevalence based on register data only as there were no measures of dementia or sleep disorders available from self-report in the full SALT cohort.

ADHD symptoms were not assessed in SALT. To evaluate if any SALT study participants had been diagnosed or treated for ADHD, we obtained information on ICD-10 ADHD diagnosis F90 from the Swedish National Patient Register and prescriptions of any ADHD-specific drug in the Prescribed Drug Register (ATC codes N06BA04, N06BA01, N06BA02, N06BA09, N06BA12). We had access to linked register data until December 31, 2013.

### Life-course risk factors

We identified four life-course risk factors previously associated with ADHD [11] as potential effect moderators and/or mediators. These included highest educational attainment (categorized as basic education, corresponding to 7 years or “folkskola”, or more than basic education, reflecting the education level distribution in the sample) [35], highest ever BMI (calculated as weight in kilograms divided by height in meters-squared [kg/m^2^], mean centered and scaled), tobacco use (ever/never for snuff and cigarettes), and alcohol misuse (defined as answering yes to one or more out of three questions concerning problematic alcohol use). For alcohol misuse, we also included information regarding lifetime alcohol use disorder from the NPR (ICD-8 304; ICD-9 291,303,305; ICD-10 F1). Life-course risk factors were assessed by self-report in the SALT interview and asked in a lifetime perspective.

Data collections in SALT/TwinGene and linkage to national registers were approved by the Swedish Ethical Review Authority in Stockholm. Informed consent was obtained from all participants.

### Statistical analyses

We estimated associations between ADHD-PRS and each somatic health outcome separately using generalized estimating equations (GEE) implemented in the R-package DrGEE [36], and adjusted the standard errors for the non-independence of twin data using a sandwich estimator. Associations are presented as odds ratios (OR) with 95% confidence interval (CIs). To assess the predictive ability of the ADHD-PRS, Nagelkerke pseudo-*R*^2^ was estimated for the full model (all covariates including ADHD-PRS) and the null model (not including ADHD-PRS). Percentage variance explained by ADHD-PRS for each outcome was calculated as the difference between the two (Δ*R*^2^). To evaluate differential associations by ascertainment source, we ran sensitivity analyses for self-reported and register-based outcomes separately. Further, we explored potential sex differences in the association of ADHD-PRS with each somatic outcome by running sex stratified analyses.

We tested whether the four life-course risk factors (i.e., education, BMI, tobacco use and alcohol misuse) were associated with ADHD-PRS, using the same statistical model as above. As all factors showed evidence of association, we further tested for moderation and mediation effects by each life-course risk factors on the ADHD-PRS-outcome associations. Moderation effects were tested by including an interaction term to the main analyses (i.e., using the same statistical models outlines above) between ADHD-PRS and each life-style risk factors, in separate models. Finally, we ran a regression-based causal mediation analysis using the counterfactual approach [37] to explore the direct and indirect effects of ADHD-PRS on somatic health outcomes mediated through education, BMI, tobacco use and alcohol misuse, separately. A directed acyclic graph representing the mediation model is provided in Supplementary Fig. S3. A bootstrap analysis with 5,000 replications with replacement was used to estimate the total effect of ADHD-PRS on an outcome, as well as the pure natural indirect effect (i.e., effect of ADHD-PRS holding constant the direct effect at the mean level), the natural direct effect (i.e., effect of ADHD-PRS holding constant the mediator at a naturally-observed value if that participant had a mean ADHD-PRS), together with their 95% CIs, and the proportion mediated (i.e., ratio of the logit for the indirect effect to the logit for the total effect). Complete mediation is indicated when the effect of ADHD-PRS on the somatic outcome (direct effect) is no longer significant after accounting for the mediating life-style risk factor, and partial mediation when this path is reduced in magnitude but remain significantly different from zero. For an in-depth definition of causal mediation effects in the counterfactual framework see refs. [38, 39]. As the life-course risk factors were assessed in a lifetime perspective, they did not necessarily proceed the outcomes. Any observed mediation effects should therefore be cautiously interpreted.

All analyses were adjusted for birth year, sex, the first six PCs, and length of follow-up in register data (i.e., years since completing the SALT interview until death, emigration, or December 31, 2013, whichever came first). We obtained false-discovery rate (FDR) corrected *p*-values using the Benjamini–Hochberg method to safeguard against multiple testing within each set of analyses. Data management was performed using SAS software version 9.4 (SAS Institute Inc., Cary, NC) and analyses ran using R 4.1.0 [40].

## Results

Among the 10,645 included individuals, 5587 (53%) were female. The mean age was 59 years (range 42–88) at self-report data collection, and 72 years (range 53–100) at the end of register-based follow-up (31st December 2013). In total, 1016 (10%) individuals had died and 9 (0.1%) emigrated after donating DNA in the TwinGene data collection (2004–2008) and before the end of register-based follow-up. Only 11 individuals had an ADHD diagnosis and/or an ADHD medication dispensation in register data, in line with evidence that ADHD is often underdiagnosed and untreated in older adult populations [5]. We kept these individuals in subsequent analyses as their inclusion is unlikely to have a strong impact on the results given the very low *N*. The number of cases and prevalence rates for somatic health outcomes are presented in Table 1 by ascertainment source.

### ADHD-PRS associations with somatic health outcomes

**Fig. 1:**
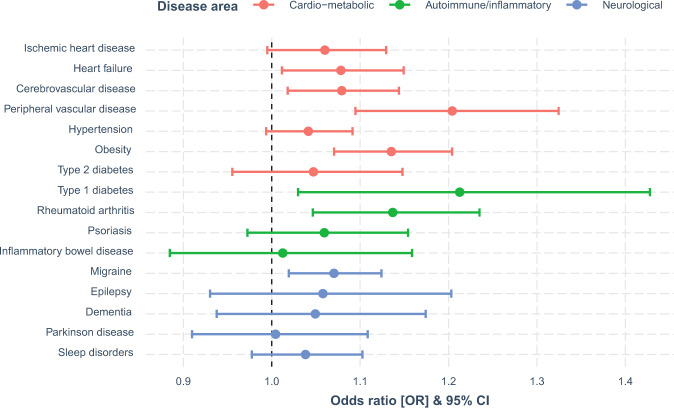
Associations of ADHD-PRS and somatic health outcomes. Present associations between ADHD-PRS and somatic health outcomes defined by combined register and self-reported data. Associations by each ascertained source separately are presented in Fig. S1. PRS, polygenic risk score. OR, odds ratio. CI, confidence interval.

Associations between ADHD-PRS and somatic health outcomes defined by combined register and self-reported data are presented Fig. 1, showing the change in risk of each outcome associated with a one SD increase of the ADHD-PRS. Of the 16 investigated somatic outcomes, seven were positively associated with ADHD-PRS after FDR correction. Point estimates, 95% CIs, FDR corrected *p*-values and Δ*R*^2^ are presented in Table 2. In the cardiometabolic domain, associations were observed for 4/7 outcomes: heart failure (OR = 1.08, 95% CI = 1.01–1.15), cerebrovascular disease (OR = 1.08, 95% CI = 1.02–1.14), peripheral vascular disease (OR = 1.20, 95% CI = 1.09–1.32), and obesity (OR = 1.14, 95% CI = 1.07–1.20). In the autoimmune/inflammatory domain, associations were observed for type 1 diabetes (OR = 1.21, 95% CI = 1.03–1.43) and rheumatoid arthritis (OR = 1.14, 95% CI = 1.05–1.23). In the neurological domain, ADHD-PRS was only associated with migraine (OR = 1.07, 95% CI = 1.02–1.12). Associations for register-based and self-reported outcomes were similar in terms of effect size (Supplementary Fig. S1), and only obesity, type 1 diabetes, rheumatoid arthritis, psoriasis, and migraine differed with regards to significance. Δ*R*^2^ for the combined, register-based, and self-reported outcomes can be found in Fig. S2. Association of ADHD-PRS with each somatic outcomes were similar in males and females, with no evidence of statistically significant sex differences (Table S4).

**Table 2:** Association of ADHD-PRS with somatic health outcomes (*N* = 10,645).

Disease area/outcome	OR (95% CI)	*p*_adj_	Δ*R*^2^ (%)
*Cardio-metabolic*			
Ischemic heart disease	1.06 (0.99–1.13)	0.142	0.059
Heart failure	**1.08** (**1.01**–**1.15)**	**0.047**	0.100
Cerebrovascular disease	**1.08** (**1.02**–**1.14)**	**0.033**	0.110
Peripheral vascular disease	**1.20** (**1.09**–**1.32)**	**0.001**	0.465
Hypertension	1.04 (0.99–1.09)	0.160	0.044
Obesity	**1.14** (**1.07**–**1.20)**	**0.000**	0.347
Type 2 Diabetes	1.05 (0.96–1.15)	0.434	0.030
*Autoimmune/inflammatory disease*			
Type 1 Diabetes	**1.21** (**1.03**–**1.43)**	**0.047**	0.379
Rheumatoid arthritis	**1.14** (**1.05**–**1.23)**	**0.013**	0.240
Psoriasis	1.06 (0.97–1.15)	0.298	0.050
Inflammatory bowel disease	1.01 (0.88–1.16)	0.914	0.002
*Neurology*			
Migraine	**1.07** (**1.02**–**1.12)**	**0.026**	0.110
Epilepsy	1.06 (0.93–1.20)	0.459	0.035
Dementia	1.05 (0.94–1.17)	0.459	0.026
Parkinson disease	1.00 (0.91–1.11)	0.932	0.000
Sleep disorder	1.04 (0.98–1.10)	0.324	0.027

Table 2 present associations between ADHD-PRS and somatic health outcomes defined by combined register and self-reported data. Associations by each ascertain source separately are presented in Fig. S1. Bolded estimates display significant false-discovery rate adjusted *p*-values.

*PRS* polygenic risk score, *OR* odds ratio, *CI* confidence interval, *P*_adj_ false-discovery rate adjusted *p*-values. Δ*R*^2^ difference in Nagelkerke pseudo-*R*^2^ expressing the percentage variance explained by ADHD-PRS in the outcome.

Descriptions of the four life-course risk factors and their associations with ADHD-PRS are presented in Table S2. Higher ADHD-PRS were associated with lower educational attainment, being underweight (BMI < 18.5) and overweight (BMI > 25) compared to BMI considered in the normal weight range (18.5–24.9), and higher risk of tobacco use and alcohol misuse.

### Moderation

No interaction effects survived multiple testing correction. Results from the moderation analyses are presented in Table 3.

**Table 3:** Moderation of ADHD-PRS associations with somatic health outcomes by education, BMI, tobacco use, and alcohol misuse.

Disease area/outcome	Education level		BMI		Tobacco use			Alcohol misuse	
	OR (95% CI)	*p*_adj_	OR (95% CI)	*p*_adj_	OR (95% CI)	*p*_adj_	OR (95% CI)		*p*_adj_
*Cardio-metabolic*									
Ischemic heart disease	1.03 (0.91–1.17)	0.969	0.97 (0.91–1.02)	0.824	1.20 (1.06–1.36)	0.779	1.14 (0.94–1.39)		0.139
Heart failure	1.02 (0.90–1.15)	0.969	1.01 (0.95–1.07)	0.969	1.11 (0.97–1.26)	0.824	0.89 (0.74–1.08)		0.724
Cerebrovascular disease	1.02 (0.91–1.14)	0.969	1.00 (0.95–1.06)	0.969	1.04 (0.92–1.17)	0.969	1.07 (0.89–1.29)		0.969
Peripheral vascular disease	0.92 (0.77–1.11)	0.969	1.01 (0.93–1.09)	0.969	1.22 (0.99–1.50)	0.969	0.97 (0.76–1.23)		0.407
Hypertension	1.01 (0.92–1.11)	0.969	0.94 (0.90–0.99)	0.167	1.04 (0.95–1.14)	0.263	0.85 (0.74–0.98)		0.969
Obesity	1.01 (0.90–1.13)	0.969	0.02 (0.00–3.17)	0.724	0.96 (0.85–1.08)	0.832	0.91 (0.77–1.07)		0.969
Type 2 Diabetes	1.02 (0.86–1.22)	0.969	0.90 (0.84–0.97)	0.139	1.01 (0.85–1.21)	0.969	0.90 (0.70–1.17)		0.969
*Autoimmune/inflammatory disease*									
Type 1 Diabetes	0.90 (0.65–1.25)	0.969	1.00 (0.89–1.12)	0.987	0.94 (0.66–1.33)	0.969	0.86 (0.57–1.29)		0.969
Rheumatoid arthritis	1.01 (0.86–1.19)	0.969	0.98 (0.90–1.06)	0.969	1.22 (1.04–1.44)	0.286	0.79 (0.64–0.98)		0.167
Psoriasis	0.79 (0.66–0.94)	0.139	0.97 (0.91–1.05)	0.969	1.04 (0.86–1.25)	0.969	0.95 (0.70–1.29)		0.969
Inflammatory bowel disease	0.97 (0.74–1.27)	0.969	1.11 (0.96–1.27)	0.778	1.07 (0.81–1.40)	0.931	0.81 (0.52–1.25)		0.969
*Neurology*									
Migraine	1.04 (0.94–1.14)	0.969	0.98 (0.93–1.02)	0.931	1.01 (0.92–1.12)	0.969	1.01 (0.85–1.19)		0.969
Epilepsy	0.94 (0.73–1.22)	0.969	1.03 (0.94–1.14)	0.969	1.18 (0.91–1.53)	0.724	1.27 (0.93–1.73)		0.824
Dementia	0.98 (0.78–1.23)	0.969	1.01 (0.91–1.12)	0.969	1.00 (0.80–1.24)	0.931	0.85 (0.63–1.16)		0.987
Parkinson disease	0.88 (0.72–1.07)	0.824	1.05 (0.97–1.14)	0.824	1.00 (0.82–1.22)	0.969	0.90 (0.67–1.22)		0.987
Sleep disorder	1.16 (1.03–1.30)	0.167	1.02 (0.96–1.07)	0.969	1.02 (0.90–1.16)	0.969	0.98 (0.83–1.15)		0.969

BMI was mean centered and treated as a continuous variable. *p*_adj_, false-discovery rate adjusted *p*-values.

*BMI* body mass index, *OR* odds ratio, *CI* confidence interval, *PRS* polygenic risk score.

### Mediation

Table 4 shows mediation effects (i.e., the pure natural indirect effect) that remained significant (*p* < 0.05) after FDR correction. An overview of the mediation results and FDR corrected *p*-values can be found in Table S3. Statistically significant mediation effects were observed for education on 3 of the 16 somatic outcomes, BMI on 9 of 15 outcomes (we did not test for mediation by BMI on obesity), tobacco use on 5 of 16 outcomes, and alcohol misuse on 8 of 16 outcomes. More specifically, full mediation was found for BMI and tobacco use in most outcomes, whereas alcohol misuse showed a mixed pattern of full and partial mediation, and only partial mediation was observed for education (see Table 4). The mediated proportion of the ADHD-PRS effect on somatic outcomes ranged from 4% (for educational attainment on peripheral vascular disease) to 27% (for BMI on cerebrovascular disease). Mediated (pure natural indirect effect) effect sizes were small, with ORs ranging from 1.003–1.052.

**Table 4:** Significant mediation effects (*p*-value < 0.05 after FDR correction) of education, BMI, tobacco use, and alcohol misuse in the associations of ADHD-PRS with somatic outcomes (*N* = 10,645).

Mediator	Somatic outcome	Mediated proportion of ADHD-PRS effect on somatic outcomes	Pure natural indirect effect OR (95% CI)	Natural direct effect OR (95% CI))	Total effect OR (95% CI)
Education	Cerebrovascular disease	0.049	1.004 (1.001–1.007)	1.074 (1.015–1.134)	1.078 (1.019–1.139)
	Peripheral vascular disease	0.037	1.006 (1.002–1.012)	1.199 (1.092–1.314)	1.207 (1.099–1.323)
	Obesity	0.046	1.006 (1.002–1.010)	1.129 (1.067–1.194)	1.135 (1.072–1.201)
BMI	Ischemic heart disease	0.112^a^	1.006 (1.001–1.011)	1.051 (0.985–1.120)	1.058 (0.991–1.127)
	Heart failure	0.168^a^	1.012 (1.007–1.018)	1.063 (0.997–1.133)	1.076 (1.009–1.147)
	Cerebrovascular disease	0.266^a^	1.020 (1.014–1.027)	1.058 (1.001–1.118)	1.079 (1.019–1.139)
	Peripheral vascular disease	0.099	1.017 (1.009–1.025)	1.178 (1.071–1.291)	1.197 (1.089–1.312)
	Hypertension	0.773^a^	1.032 (1.023–1.042)	1.009 (0.964–1.056)	1.042 (0.995–1.091)
	Type 2 diabetes	0.600^a^	1.052 (1.037–1.068)	0.976 (0.894–1.066)	1.026 (0.940–1.121)
	Type 1 diabetes	0.160^a^	1.027 (1.016–1.039)	1.163 (0.989–1.370)	1.194 (1.015–1.407)
	Migraine	0.074	1.005 (1.002–1.009)	1.067 (1.018–1.118)	1.072 (1.024–1.123)
	Sleep disorders	0.408^a^	1.016 (1.011–1.023)	1.024 (0.967–1.087)	1.041 (0.982–1.104)
Tobacco	Cerebrovascular disease	0.102^a^	1.007 (1.004–1.012)	1.070 (1.012–1.131)	1.078 (1.019–1.140)
	Peripheral vascular disease	0.091	1.017 (1.009–1.025)	1.199 (1.090–1.314)	1.219 (1.108–1.337)
	Type 2 diabetes	0.117^a^	1.005 (1.001–.010)	1.041 (0.955–1.134)	1.046 (0.960–1.141)
	Psoriasis	0.105^a^	1.006 (1.002–.011)	1.056 (0.969–1.149)	1.062 (0.975–1.156)
	Sleep disorders	0.226^a^	1.008 (1.004–1.013)	1.030 (0.972–1.094)	1.038 (0.979–1.103)
Alcohol	Ischemic heart disease	0.083^a^	1.005 (1.001–1.009)	1.057 (0.991–1.126)	1.062 (0.996–1.132)
	Heart failure	0.066^a^	1.005 (1.001–1.009)	1.073 (1.007–1.144)	1.078 (1.011–1.148)
	Cerebrovascular disease	0.052	1.004 (1.001–1.008)	1.074 (1.015–1.134)	1.078 (1.019–1.139)
	Peripheral vascular disease	0.046	1.008 (1.002–1.016)	1.198 (1.090–1.313)	1.208 (1.099–1.324)
	Hypertension	0.074^a^	1.003 (1.001–1.006)	1.039 (0.994–1.085)	1.042 (0.997–1.088)
	Obesity	0.033	1.004 (1.001–1.008)	1.130 (1.069–1.195)	1.135 (1.072–1.201)
	Type 2 diabetes	0.087^a^	1.004 (1.001–1.009)	1.041 (0.957–1.134)	1.045 (0.961–1.139)
	Sleep disorders	0.203	1.008 (1.002–1.015)	1.032 (0.974–1.096)	1.041 (0.982–1.105)

Table 4 present the total effect, pure natural indirect, and natural direct effects, and the proportion mediated (i.e., ratio of the logit for the indirect effect to the logit for the total effect) by each mediator for each somatic health outcome. Associations are expressed as odds ratios together with their 95% CIs and the proportion mediated as the proportion of the total effect that is mediated by each life-style risk factor. BMI was mean centered and treated as a continuous variable.

Results are only shown here for mediation effects (i.e., the pure natural indirect effect) that remained significant (*p* < 0.05) after false-discovery rate correction. Full results for all mediation effects together with FDR-corrected *p*-values are presented in Table S3.

*PRS* polygenic risk score, *OR* odds ratio, *CI* confidence interval, *p*_adj,_ false-discovery rate (FDR) adjusted *p*-values, *BMI* body mass index.

^a^Displays full mediation, the remaining effects are partial mediations.

## Discussion

We evaluated the role of ADHD genetic liability, as captured by PRS, on somatic health conditions in mid-to-late life in a general population sample. Our results suggest that polygenic risk for ADHD confers modestly increased risks for a range of somatic health problems in adulthood, particularly in the cardiometabolic domain. These associations were evident in a general population sample untreated and undiagnosed for ADHD. Further, we present novel, albeit tentative, findings to suggest that part of the association between ADHD genetic liability and primarily cardiometabolic outcomes may be mediated by life-course risk factors (i.e., education, BMI, tobacco use and alcohol misuse).

Higher ADHD-PRS were associated with four out of seven cardiometabolic outcomes, including obesity, peripheral vascular disease, heart failure and cerebrovascular disease. There is an extensive body of literature linking ADHD to obesity [31]. Further, obesity-related phenotypes represent some of the strongest GWAS-based genetic correlations with ADHD outside the psychiatric domain [9, 21, 41]. A recent study implicated genes upregulated in the reward system in the genetic link between ADHD and BMI [41]. Further, neuroimaging studies suggest that a common neural substrate related to reward processing may mediate the shared genetic liability for ADHD and BMI, and their phenotypic association [42]. Whilst we found that the ADHD-PRS associations with type 2 diabetes, ischemic heart disease, hypertension, were positive, they did not survive multiple testing correction, potentially due to low power in our sample or because of relatively weaker genetic links to ADHD. GWAS-based genetic correlation have previously been reported of ADHD with type 2 diabetes and coronary artery disease [9], however, we are not aware of studies replicating such findings using PRS.

Overall, we found less evidence for genetic links between ADHD-PRS and diseases in the autoimmune/inflammatory and neurological domains. Among the autoimmune/inflammatory diseases, ADHD-PRS were associated with a small increased risk for type 1 diabetes and rheumatoid arthritis, but not psoriasis or inflammatory bowel disease. Some prior studies have found associations of clinical ADHD with type 1 diabetes and rheumatoid arthritis, both within-individual and across relatives, suggesting a possible common genetic vulnerability between ADHD and certain autoimmune diseases [43, 44]. However, the association with rheumatoid arthritis was not replicated in a recent large-scale Swedish register-based study [45]. GWAS-based genetic correlations of ADHD with rheumatoid arthritis and psoriasis (*r*_g_ 0.16 and 0.23, respectively) were reported in one prior study, whereas the association to Type 1 diabetes was not found to be statistically significant [8]. More research is thus warranted to understand if and how potential shared genetic risk, neuro-inflammatory pathways, and life-course risk factors may connect ADHD to autoimmune/inflammatory disease.

Within the neurological domain, migraine was the only outcome associated with ADHD-PRS, with no significant associations observed for epilepsy, dementia, Parkinson disease, or sleep disorders. Although there are well-established phenotypic links between ADHD and certain neurological diseases (e.g., epilepsy, migraine, sleep disorders), evidence of genetic overlap with ADHD are mixed [7]. Large-scale GWAS analyses thus far provide limited evidence for genetic correlations of ADHD with epilepsy and neurodegenerative disorders, whereas migraine and sleep related phenotypes are positively and significant genetically correlated with ADHD at the level of common variants (*r*_g_ ~ 0.2 to 0.4) [10, 46]. A quantitative genetic family study also found that whilst the association between ADHD and migraine appears to be almost entirely explained by genetic factors, associations with sleep disorders and epilepsy were to a larger extent explained by environmental factors [45]. Together, this suggests that comorbidity of ADHD with e.g., migraine may arise from common genetic pleiotropic effects, whereas neurological conditions like epilepsy and sleep disorders may in part be linked with ADHD through direct causal effects, or environmentally mediated effects. It is also possible that the lack of strong evidence for a genetic association of ADHD with sleep disorder in the current analyses and the aforementioned quantitative genetic analyses [45], in part reflect the relatively poor coverage and high degree of misclassification of sleep disorders in Swedish national register data. Research on ADHD and neurodegenerative disorders, including dementia, are still scarce, precluding strong conclusions about their potential shared etiology [7].

We found no evidence for moderation of ADHD-PRS associations by the investigated life-course risk factors (i.e., education, BMI, tobacco use, and alcohol misuse), with minor differences observed between exposure groups. The few available studies investigating interaction effects using ADHD-PRS have focused on ADHD as the outcome and largely reported null findings [20], much like PRS interaction testing other areas in medicine [47]. Similarly, we found no evidence of sex differences in the associations of ADHD-PRS with somatic outcomes. This in line with prior reports in UKBiobank [22] and a recent large-scale family-based register study [45], neither of which found strong support for sex differences. Nevertheless, detecting interaction effect using PRS is challenging and will likely require large, well-powered samples. Further, disorder predictive PRSs may not necessarily capture genetic variants linked to a differential susceptibility to risk-factors exposure [48]. This could potentially explain why no moderation effects survived multiple testing correction in this study.

In contrast, we did find support for a mediating role of lower education, higher BMI, tobacco use, and alcohol misuse, on the relationship between ADHD-PRS and several somatic outcomes. Mediation effects were observed for nearly all cardiometabolic outcomes, for multiple mediators. For example, the main effect of ADHD-PRS on peripheral- and cardiovascular diseases showed significant mediation by all four life-course risk-factors. Further, BMI and alcohol misuse showed evidence of mediation on all significant ADHD-PRS main effects in the cardiometabolic domain (i.e., heart failure, ischemic and peripheral hearth disease). Further, BMI also mediated part of the association of higher ADHD-PRS with type 1 diabetes and migraine. The mediating effect of higher BMI across disease domain could either reflect the broad systemic effect that obesity can have on health and the strong link between ADHD and obesity. It may also in part be because BMI was the only mediator modeled as a continuous variable, thus increasing the power to detect even very small mediation effects. It is important to note that the life-course risk factors were asked about in a lifetime manner, meaning they did not necessarily precede the outcomes and mediation effects should therefore be cautiously interpreted. Further, it is important to note that both the main and mediation effects of ADHD-PRS were modest across all three disease domains, and our findings are thus far from clinical utility (e.g., risk-screening). This is unsurprising given that the current ADHD-PRS only explains about 5% of variance in ADHD diagnosis, and considerably less in related traits and comorbidities [9, 20]. However, it does suggest that our findings may not reflect the total genetic overlap between ADHD and mid-to-late life somatic health. If our findings of mediation effects are replicated in independent samples with clear temporal ordering, it would suggest that at least some of the reported genetic associations between ADHD and worse cardiometabolic health may be due to mediated genetic pleiotropy that acts via potentially modifiable life-course risk factors, including higher BMI. Such an interpretation complements findings from a recent Mendelian Randomization study, which reported a bidirectional relationship between ADHD and childhood obesity, whereas the observed ‘causal’ effect of ADHD on coronary artery disease attenuated when controlling for obesity [49].

Our results add to a growing body of evidence suggesting that ADHD common variant genetic liability confers a small increased risk for several mid-to-late life somatic health condition [21, 22]. Moreover, these associations were evident in a general population sample where the vast majority were untreated for ADHD (only 11 individuals had been diagnosed or treated for ADHD). This suggests the increased risk for worse somatic health outcomes (e.g., cardiometabolic risk, hypertension) in ADHD cannot merely be attributed to negative effects of ADHD drug treatment and are likely present in individuals with subclinical ADHD and/or higher ADHD genetic burden. Together, these findings warrant further research and clinical focus on the long-term health of individuals with elevated ADHD symptoms both above and below an arbitrarily defined clinical threshold.

### Strengths and limitations

There are several strengths but also important limitations of this study. First, assessment of somatic health outcomes by both self-report and national medical registers represents an advantage of this study, yet the two data sources may capture partly different case definitions. Self-report provides information on somatic health problems that are untreated or mostly treated in primary care (which we did not have information from), and for symptoms below the diagnostic threshold. Conversely, register data from specialist care captures more severely affected patients and has excellent coverage for diseases requiring specialist treatment (e.g., acute cardiovascular events, epilepsy, Parkinson disease) [32]. As such, we observed some variation in associations across data source. For example, ADHD-PRS were only significantly associated with self-reported, but not register-based, rheumatoid arthritis, obesity, and migraine. As these conditions are generally treated in primary care, coverage in the register data (Table 1) is poor, potentially explaining differential associations across self-report and register-based definitions. Second, self-report of somatic health conditions and life-course risk factors were collected cross-sectional, meaning we were unable to study potentially important age effects, and that any mediation results must be interpreted with caution. Third, the SALT cohort includes individuals who have volunteered to participate in several data collections, leading to potential participation bias. Indeed, higher ADHD genetic liability has previously been negatively associated with study participation [50]. As such, the any observed associations observed were likely attenuated compared to those in the background population and in clinical ADHD samples. Finally, the SALT cohort is a highly homogenous European ancestry population sample, which may limit generalizability to more diverse and clinical populations.

## Conclusion

Our study suggests an overall pattern whereby higher ADHD genetic liability is linked to a modest risk increase for several somatic health problems in adult life, particularly in the cardiometabolic domain. These associations were observable in the general population, even in the absence of medical treatment for ADHD, and may in part be mediated via life-course risk factors.

## Supplementary information

Supplementary materials
