# Correction: The role of ADHD genetic risk in mid-to-late life somatic health conditions

**DOI:** 10.1038/s41398-022-01933-x

**Published:** 2022-04-21

**Authors:** Miguel Garcia-Argibay, Ebba du Rietz, Yi Lu, Joanna Martin, Elis Haan, Kelli Lehto, Sarah E. Bergen, Paul Lichtenstein, Henrik Larsson, Isabell Brikell

**Affiliations:** 1 School of Medical Sciences, Örebro University, Örebro, Sweden; 2 Department of Medical Epidemiology and Biostatistics, Karolinska Institutet, Stockholm, Sweden; 3 MRC Centre for Neuropsychiatric Genetics and Genomics, Cardiff University, Cardiff, UK; 4 Estonian Genome Centre, Institute of Genomics, University of Tartu, Tartu, Estonia

**Keywords:** ADHD, Clinical genetics

Correction to: *Translational Psychiatry* 10.1038/s41398-022-01919-9, published online 11 April 2022

The original version of this article unfortunately contained a mistake in an author name. The correct name is Kelli Lehto. The authors apologize for the error. The original article has been corrected.

