# Supplementary material for: The role of ADHD genetic risk in mid-to-late life somatic health conditions
Source: Transl Psychiatry. 2022 Apr 11;12:152. doi: 10.1038/s41398-022-01919-9 (PMC8995388; doi:10.1038/s41398-022-01919-9)
Supplement: Supplementary file 1 — Supplementary materials [file 41398_2022_1919_MOESM1_ESM.docx]

**Supplementary materials**

**The role of ADHD genetic risk in mid-to-late life somatic health conditions**

**Running title: ADHD genetics and mid-to-late life somatic health**

Miguel Garcia-Argibay^1,2^, Ph.D., Ebba du Rietz^2^, Ph.D., Yi Lu^2^, PhD, Joanna Martin^2,3^, Elis Haan^4^, Ph.D., Kelli Letho^4^, Ph.D., Sarah E. Bergen^2^, Ph.D., Paul Lichtenstein^2^, Ph.D., Henrik Larsson^1,2^, Ph.D., & Isabell Brikell^2*^, Ph.D.

^1^ School of Medical Sciences, Örebro University, Örebro, Sweden

^2^ Department of Medical Epidemiology and Biostatistics, Karolinska Institutet, Stockholm, Sweden

^3^ MRC Centre for Neuropsychiatric Genetics and Genomics, Cardiff University, Cardiff, UK

^4^ Estonian Genome Centre, Institute of Genomics, University of Tartu, Tartu, Estonia

Content

[Supplementary note. SALT/TwinGene basic quality control and imputation 2](#_Toc95903277)

[Table S1. ICD and ATC codes for register-based definitions of somatic health problems 3](#_Toc95903278)

[Table S2. N, % prevalence and association of ADHD-PRS with potential effect modifiers/mediators 5](#_Toc95903279)

[Table S3. Mediation effect of education, BMI, tobacco and alcohol misuse in the associations of ADHD-PRS with somatic outcomes (N=10,645) 6](#_Toc95903280)

[Table S4. Moderation effect of sex in the associations of ADHD-PRS with somatic outcomes (N=10,645) 12](#_Toc95903281)

[Figure S1. Associations of ADHD-PRS and somatic health outcomes evaluated by register-data and self-reported compared to the combined estimates, expressed by standard deviation of the PRS 13](#_Toc95903282)

[Figure S2. NagelKerke pseudo-R^2^ for the associations of ADHD-PRS and somatic health outcomes evaluated by register-data and self-reported compared to the combined estimates, expressed by standard deviation of the PRS 14](#_Toc95903283)

[Figure S3. Directed acyclic graph illustrating the mediation models 15](#_Toc95903284)

# Supplementary note. SALT/TwinGene basic quality control and imputation

Data collection, DNA extraction and genotyping procedures in TwinGene has been described in detail elsewhere.^1^ Genotyping results for 9836 subjects passed the initial lab-based quality control.

***Basic quality control***

Basic quality control (QC) steps of the primary genotyping data (9836 subjects and 731442 SNPs) were:

1. Marker missingness filter removed 3922 SNPs due to more than 3% missing information (GENO > 0.03).

2. Low genotyping success (MIND>0.03) removed 10 individuals.

3. Allele frequency filter removed 79893 SNPs with minor allele frequency < 1%.

4. Sex-check (heterozygosity of X-chromosomes) resulted in removal of 36 individuals.

5. Deviations from Hardy-Weinberg equilibrium at the level of *p* ≤ 1^e-07^ led to exclusions of 3071 markers.

6. 49 individuals were excluded due to deviations in heterozygosity of more than 5 standard deviations from the population mean.

7. Detection of unknown (cryptic) relatedness resulted in removal of 124 individuals.

8. After the basic QC, 9617 individuals and 644556 SNPs remained in the sample

***Imputation***

Imputation using the 1000G Phase 1 version 3 (1000G data freeze: 2010-11-23. Genotype release: 2011-05-21) as reference panel was performed by splitting each chromosome into chunks of 2500 SNPs with a 500 SNP overlap. Pre-phasing and imputation were performed separately on each chunk using MaCH/minimac1 (version 1.0.18.c, MaCH with monomorphic removed release: 2012-03-14), resulting in 13,643,174 imputed SNPs.

**References**

1. Magnusson, P.K.*, et al.* The Swedish Twin Registry: establishment of a biobank and other recent developments. *Twin Res Hum Genet* **16**, 317-329 (2013).

# Table S1. ICD and ATC codes for register-based definitions of somatic health problems

| **Somatic health outcomes** | **ICD codes** | | | **ATC codes** |
| --- | --- | --- | --- | --- |
| **Cardio-metabolic** | **ICD-8** | **ICD-9** | **ICD-10** |  |
| Ischemic heart disease | 410-414 | 410-414 | I20-I25 |  |
| Heart failure | 427.00, 427.19, 427.99 | 428 | I50 |  |
| Cerebrovascular disease | 356.00-356.09, 430,431,432, 433,434,436,437.99, 438.99, 440.20 | 344W, 352G, 430,431,432, 433, 434,436, 437 | I60-I69, G45, G46 |  |
| Peripheral vascular disease | 421.00, 440, 441, 442, 443, 444, 447.00-09 | 421A, 440, 441, 442, 443, 444, 447 | I70-74, I77 |  |
| Hypertension | 400-404 | 401-405 | I10-I13, I15 |  |
| Obesity | 277.99 | 278A, 278B | E65-E66 |  |
| Type 2 diabetes mellitus | N/A | N/A | E11 |  |
| **Autoimmune/inflammatory** | | | | |
| Type 1 diabetes mellitus | 250 | 250 | E10 |  |
| Rheumatoid arthritis | 712.10, 712.20, 712.38, 713.39 | 714A-C, 714W, 719D | M05, M06.0, M06.2, M06.3, M06.8, M06.9, M12.3 |  |
| Psoriasis | 696 | 696 | L40 |  |
| Inflammatory Bowel Disease | 5630, 5631, 569 | 555, 556 | K50, K51 |  |
| **Neurological** | | | | |
| Migraine | 346.09 | 346A/B/X/W | G43 | NO2CC |
| Epilepsy | 345 | 345 | G40-41 |  |
| Dementia (any) | 290.00, 290.10, 290.11, 290.19, 293.00, 293.1 | 290A, 290B, 290E, 290W, 290X, 294B, 331A, 331B, 331C, 331X | G30, G31.1, G31.8A, F00-F03, F05.1 | N06D |
| Parkinson disease and parkinsonism | 342.00, 342.08, 342.09 | 332A | G20, G21.2, G21.3, G21.8, G21.9, G23.1, G23.2, G23.8, G23.9, G25.9 | N04B |
| Sleep disorder | 780.6, 306.4 | 780F | G47.0, G47.1, G47.2, G47.3, G47.8, G47.9 | N05C |

# Table S2. N, % prevalence and association of ADHD-PRS with potential effect modifiers/mediators

| **Moderator/Mediator** | **n (%)** | **OR (95% CI)** | ***p*** |
| --- | --- | --- | --- |
| Education > 7yrs | 6084 (57.2) | 0.93 (0.89-0.97) | < .001 |
| BMI (%) |  |  |  |
| *<18.5 kg/m^2^* | 20 (0.2) | 1.08 (1.08-1.09) | < .001 |
| *18.5–24.9 kg/m^2^* | 4045 (38.7) | Reference |  |
| *25.0–29.9 kg/m^2^* | 4856 (46.5) | 1.09 (1.05-1.14) | < .001 |
| *≥30 kg/m^2^* | 1526 (14.6) | 1.19 (1.12-1.26) | < .001 |
| Tobacco use | 6246 (59.1) | 1.10 (1.06-1.14) | < .001 |
| Alcohol misuse | 900 (8.5) | 1.10 (1.03-1.19) | 0.006 |

**Abbreviations**: BMI, body mass index. PRS, polygenic risk score. OR, odds ratio. CI, confidence interval.

# Table S3. Mediation effect of education, BMI, tobacco and alcohol misuse in the associations of ADHD-PRS with somatic outcomes (N=10,645)

| **Mediator** | **Outcome** | **Effect** | **Mediated proportion /**  **OR (95% CI)** | ***p*_adj_** |
| --- | --- | --- | --- | --- |
| Education | Ischemic heart disease | Proportion mediated | 0.008 | 0.834 |
|  |  | Pure natural indirect effect | 1.000 (0.998-1.003) | 0.821 |
|  |  | Natural direct effect | 1.061 (0.995-1.130) | 0.163 |
|  |  | Total effect | 1.061 (0.996-1.130) | 0.163 |
| Education | Heart failure | Proportion mediated | 0.003 | 0.929 |
|  |  | Pure natural indirect effect | 1.000 (0.998-1.002) | 0.929 |
|  |  | Natural direct effect | 1.077 (1.011-1.147) | 0.071 |
|  |  | Total effect | 1.077 (1.011-1.147) | 0.071 |
| Education | Cerebrovascular disease | Proportion mediated | 0.049 | 0.043 |
|  |  | Pure natural indirect effect | **1.004 (1.001-1.007)** | 0.013 |
|  |  | Natural direct effect | 1.074 (1.015-1.134) | 0.044 |
|  |  | Total effect | 1.078 (1.019-1.139) | 0.036 |
| Education | Peripheral vascular disease | Proportion mediated | 0.037 | 0.013 |
|  |  | Pure natural indirect effect | **1.006 (1.002-1.012)** | 0.013 |
|  |  | Natural direct effect | 1.199 (1.092-1.314) | <0.001 |
|  |  | Total effect | 1.207 (1.099-1.323) | <0.001 |
| Education | Hypertension | Proportion mediated | 0.015 | 0.625 |
|  |  | Pure natural indirect effect | 1.001 (0.999-1.002) | 0.593 |
|  |  | Natural direct effect | 1.041 (0.996-1.087) | 0.171 |
|  |  | Total effect | 1.042 (0.997-1.088) | 0.163 |
| Education | Obesity | Proportion mediated | 0.046 | 0.013 |
|  |  | Pure natural indirect effect | **1.006 (1.002-1.010)** | 0.013 |
|  |  | Natural direct effect | 1.129 (1.067-1.194) | <0.001 |
|  |  | Total effect | 1.135 (1.072-1.201) | <0.001 |
| Education | Type 2 diabetes | Proportion mediated | 0.068 | 0.531 |
|  |  | Pure natural indirect effect | 1.003 (0.999-1.007) | 0.136 |
|  |  | Natural direct effect | 1.042 (0.958-1.135) | 0.529 |
|  |  | Total effect | 1.046 (0.961-1.139) | 0.505 |
| Education | Type 1 diabetes | Proportion mediated | 0.020 | 0.372 |
|  |  | Pure natural indirect effect | 1.003 (0.998-1.010) | 0.361 |
|  |  | Natural direct effect | 1.206 (1.029-1.419) | 0.060 |
|  |  | Total effect | 1.210 (1.032-1.426) | 0.054 |
| Education | Rheumatoid arthritis | Proportion mediated | 0.029 | 0.061 |
|  |  | Pure natural indirect effect | 1.003 (1.000-1.008) | 0.06 |
|  |  | Natural direct effect | 1.132 (1.041-1.230) | 0.019 |
|  |  | Total effect | 1.136 (1.045-1.234) | 0.013 |
| Education | Psoriasis | Proportion mediated | 0.020 | 0.675 |
|  |  | Pure natural indirect effect | 1.001 (0.998-1.004) | 0.596 |
|  |  | Natural direct effect | 1.058 (0.972-1.152) | 0.353 |
|  |  | Total effect | 1.059 (0.973-1.154) | 0.345 |
| Education | Inflammatory bowel disease | Proportion mediated | 0.006 | 0.999 |
|  |  | Pure natural indirect effect | 1.000 (0.995-1.005) | 0.995 |
|  |  | Natural direct effect | 1.012 (0.887-1.159) | 0.929 |
|  |  | Total effect | 1.012 (0.887-1.159) | 0.929 |
| Education | Migraine | Proportion mediated | 0.005 | 0.826 |
|  |  | Pure natural indirect effect | 1.000 (0.999-1.002) | 0.826 |
|  |  | Natural direct effect | 1.070 (1.021-1.121) | 0.027 |
|  |  | Total effect | 1.070 (1.022-1.122) | 0.027 |
| Education | Epilepsy | Proportion mediated | -0.011 | 0.943 |
|  |  | Pure natural indirect effect | 0.999 (0.994-1.004) | 0.891 |
|  |  | Natural direct effect | 1.063 (0.936-1.210) | 0.512 |
|  |  | Total effect | 1.063 (0.936-1.209) | 0.517 |
| Education | Dementia | Proportion mediated | 0.051 | 0.691 |
|  |  | Pure natural indirect effect | 1.002 (0.999-1.007) | 0.361 |
|  |  | Natural direct effect | 1.047 (0.939-1.168) | 0.593 |
|  |  | Total effect | 1.049 (0.941-1.170) | 0.569 |
| Education | Parkinson | Proportion mediated | 0.181 | 0.955 |
|  |  | Pure natural indirect effect | 1.001 (0.998-1.005) | 0.716 |
|  |  | Natural direct effect | 1.004 (0.915-1.105) | 0.955 |
|  |  | Total effect | 1.005 (0.916-1.107) | 0.953 |
| Education | Sleep disorders | Proportion mediated | -0.027 | 0.608 |
|  |  | Pure natural indirect effect | 0.999 (0.997-1.001) | 0.505 |
|  |  | Natural direct effect | 1.041 (0.982-1.105) | 0.345 |
|  |  | Total effect | 1.040 (0.981-1.104) | 0.349 |
| BMI | Ischemic heart disease | Proportion mediated | 0.112 | 0.195 |
|  |  | Pure natural indirect effect | **1.006 (1.001-1.011)** | 0.044 |
|  |  | Natural direct effect | 1.051 (0.985-1.120) | 0.251 |
|  |  | Total effect | 1.058 (0.991-1.127) | 0.023 |
| BMI | Heart failure | Proportion mediated | 0.168 | 0.077 |
|  |  | Pure natural indirect effect | **1.012 (1.007-1.018)** | <0.001 |
|  |  | Natural direct effect | 1.063 (0.997-1.133) | 0.152 |
|  |  | Total effect | 1.076 (1.010-1.147) | 0.077 |
| BMI | Cerebrovascular disease | Proportion mediated | 0.266 | 0.036 |
|  |  | Pure natural indirect effect | **1.020 (1.014-1.027)** | <0.001 |
|  |  | Natural direct effect | 1.058 (1.000-1.118) | 0.125 |
|  |  | Total effect | 1.079 (1.020-1.140) | 0.036 |
| BMI | Peripheral vascular disease | Proportion mediated | 0.099 | <0.001 |
|  |  | Pure natural indirect effect | **1.017 (1.009-1.025)** | <0.001 |
|  |  | Natural direct effect | 1.178 (1.071-1.291) | 0.009 |
|  |  | Total effect | 1.197 (1.089-1.312) | <0.001 |
| BMI | Hypertension | Proportion mediated | 0.773 | 0.176 |
|  |  | Pure natural indirect effect | **1.032 (1.023-1.042)** | <0.001 |
|  |  | Natural direct effect | 1.010 (0.964-1.056) | 0.826 |
|  |  | Total effect | 1.042 (0.995-1.091) | 0.176 |
| BMI | Type 2 diabetes | Proportion mediated | 0.600 | 0.725 |
|  |  | Pure natural indirect effect | **1.052 (1.037-1.068)** | <0.001 |
|  |  | Natural direct effect | 0.976 (0.894-1.066) | 0.750 |
|  |  | Total effect | 1.026 (0.940-1.121) | 0.725 |
| BMI | Type 1 diabetes | Proportion mediated | 0.161 | 0.080 |
|  |  | Pure natural indirect effect | **1.027 (1.016-1.039)** | <0.001 |
|  |  | Natural direct effect | 1.163 (0.989-1.370) | 0.163 |
|  |  | Total effect | 1.194 (1.015-1.407) | 0.080 |
| BMI | Rheumatoid arthritis | Proportion mediated | 0.038 | 0.305 |
|  |  | Pure natural indirect effect | 1.004 (0.998-1.010) | 0.304 |
|  |  | Natural direct effect | 1.119 (1.029-1.216) | 0.035 |
|  |  | Total effect | 1.124 (1.034-1.222) | 0.024 |
| BMI | Psoriasis | Proportion mediated | 0.063 | 0.512 |
|  |  | Pure natural indirect effect | 1.004 (0.998-1.010) | 0.369 |
|  |  | Natural direct effect | 1.060 (0.973-1.155) | 0.345 |
|  |  | Total effect | 1.064 (0.978-1.160) | 0.304 |
| BMI | Inflammatory bowel disease | Proportion mediated | 0.059 | 0.943 |
|  |  | Pure natural indirect effect | 1.001 (0.989-1.011) | 0.929 |
|  |  | Natural direct effect | 1.018 (0.893-1.165) | 0.893 |
|  |  | Total effect | 1.019 (0.894-1.169) | 0.891 |
| BMI | Migraine | Proportion mediated | 0.074 | 0.031 |
|  |  | Pure natural indirect effect | **1.005 (1.002-1.009)** | 0.013 |
|  |  | Natural direct effect | 1.067 (1.018-1.118) | 0.036 |
|  |  | Total effect | 1.072 (1.024-1.123) | 0.024 |
| BMI | Epilepsy | Proportion mediated | 0.181 | 0.596 |
|  |  | Pure natural indirect effect | 1.01 (0.999-1.02) | 0.163 |
|  |  | Natural direct effect | 1.045 (0.919-1.191) | 0.652 |
|  |  | Total effect | 1.055 (0.927-1.201) | 0.569 |
| BMI | Dementia | Proportion mediated | -0.018 | 0.929 |
|  |  | Pure natural indirect effect | 0.999 (0.990-1.007) | 0.893 |
|  |  | Natural direct effect | 1.066 (0.954-1.192) | 0.439 |
|  |  | Total effect | 1.065 (0.952-1.191) | 0.450 |
| BMI | Parkinson | Proportion mediated | 0.236 | 0.891 |
|  |  | Pure natural indirect effect | 1.004 (0.998-1.011) | 0.361 |
|  |  | Natural direct effect | 1.014 (0.922-1.114) | 0.903 |
|  |  | Total effect | 1.018 (0.927-1.120) | 0.853 |
| BMI | Sleep disorders | Proportion mediated | 0.408 | 0.345 |
|  |  | Pure natural indirect effect | **1.016 (1.011-1.023)** | <0.001 |
|  |  | Natural direct effect | 1.024 (0.967-1.087) | 0.593 |
|  |  | Total effect | 1.041 (0.982-1.104) | 0.345 |
| Tobacco | Ischemic heart disease | Proportion mediated | 0.058 | 0.186 |
|  |  | Pure natural indirect effect | 1.003 (0.999-1.007) | 0.063 |
|  |  | Natural direct effect | 1.058 (0.992-1.127) | 0.179 |
|  |  | Total effect | 1.061 (0.994-1.131) | 0.163 |
| Tobacco | Heart failure | Proportion mediated | 0.017 | 0.596 |
|  |  | Pure natural indirect effect | 1.001 (0.998-1.004) | 0.587 |
|  |  | Natural direct effect | 1.073 (1.006-1.143) | 0.084 |
|  |  | Total effect | 1.074 (1.008-1.145) | 0.079 |
| Tobacco | Cerebrovascular disease | Proportion mediated | 0.102 | 0.043 |
|  |  | Pure natural indirect effect | **1.007 (1.004-1.012)** | <0.001 |
|  |  | Natural direct effect | 1.070 (1.012-1.131) | 0.061 |
|  |  | Total effect | 1.078 (1.019-1.140) | 0.043 |
| Tobacco | Peripheral vascular disease | Proportion mediated | 0.091 | <0.001 |
|  |  | Pure natural indirect effect | **1.017 (1.009-1.025)** | <0.001 |
|  |  | Natural direct effect | 1.199 (1.009-1.314) | <0.001 |
|  |  | Total effect | 1.219 (1.108-1.337) | <0.001 |
| Tobacco | Hypertension | Proportion mediated | 0.061 | 0.181 |
|  |  | Pure natural indirect effect | 1.002 (0.999-1.005) | 0.054 |
|  |  | Natural direct effect | 1.039 (0.995-1.086) | 0.189 |
|  |  | Total effect | 1.042 (0.997-1.089) | 0.163 |
| Tobacco | Obesity | Proportion mediated | 0.023 | 0.085 |
|  |  | Pure natural indirect effect | 1.003 (1.000-1.006) | 0.085 |
|  |  | Natural direct effect | 1.134 (1.072-1.200) | <0.001 |
|  |  | Total effect | 1.138 (1.075-1.204) | <0.001 |
| Tobacco | Type 2 diabetes | Proportion mediated | 0.117 | 0.505 |
|  |  | Pure natural indirect effect | **1.005 (1.001-1.010)** | 0.036 |
|  |  | Natural direct effect | 1.041 (0.955-1.134) | 0.547 |
|  |  | Total effect | 1.046 (0.960-1.141) | 0.505 |
| Tobacco | Type 1 diabetes | Proportion mediated | 0.024 | 0.429 |
|  |  | Pure natural indirect effect | 1.004 (0.997-1.013) | 0.408 |
|  |  | Natural direct effect | 1.212 (1.033-1.427) | 0.058 |
|  |  | Total effect | 1.217 (1.036-1.433) | 0.054 |
| Tobacco | Rheumatoid arthritis | Proportion mediated | 0.022 | 0.345 |
|  |  | Pure natural indirect effect | 1.003 (0.999-1.007) | 0.345 |
|  |  | Natural direct effect | 1.132 (1.040-1.203) | 0.013 |
|  |  | Total effect | 1.135 (1.043-1.233) | 0.013 |
| Tobacco | Psoriasis | Proportion mediated | 0.105 | 0.338 |
|  |  | Pure natural indirect effect | **1.006 (1.002-1.011)** | 0.009 |
|  |  | Natural direct effect | 1.056 (0.969-1.149) | 0.374 |
|  |  | Total effect | 1.062 (0.975-1.156) | 0.338 |
| Tobacco | Inflammatory bowel disease | Proportion mediated | -0.904 | 0.998 |
|  |  | Pure natural indirect effect | 1.003 (0.997-1.009) | 0.531 |
|  |  | Natural direct effect | 0.994 (0.871-1.140) | 0.969 |
|  |  | Total effect | 0.997 (0.874-1.143) | 0.994 |
| Tobacco | Migraine | Proportion mediated | -0.041 | 0.077 |
|  |  | Pure natural indirect effect | 0.997 (0.994-0.998) | 0.065 |
|  |  | Natural direct effect | 1.070 (1.022-1.122) | 0.024 |
|  |  | Total effect | 1.068 (1.019-1.119) | 0.031 |
| Tobacco | Epilepsy | Proportion mediated | 0.015 | 0.941 |
|  |  | Pure natural indirect effect | 1.001 (0.994-1.008) | 0.923 |
|  |  | Natural direct effect | 1.055 (0.928-1.198) | 0.569 |
|  |  | Total effect | 1.056 (0.928-1.199) | 0.569 |
| Tobacco | Dementia | Proportion mediated | 0.011 | 0.943 |
|  |  | Pure natural indirect effect | 1.001 (0.995-1.006) | 0.929 |
|  |  | Natural direct effect | 1.049 (0.940-1.170) | 0.569 |
|  |  | Total effect | 1.050 (0.940-1.171) | 0.569 |
| Tobacco | Parkinson | Proportion mediated | -0.041 | 0.994 |
|  |  | Pure natural indirect effect | 1.000 (0.995-1.004) | 0.943 |
|  |  | Natural direct effect | 1.008 (0.918-1.110) | 0.940 |
|  |  | Total effect | 1.008 (0.918-1.110) | 0.942 |
| Tobacco | Sleep disorders | Proportion mediated | 0.226 | 0.361 |
|  |  | Pure natural indirect effect | **1.008 (1.004-1.013)** | 0.001 |
|  |  | Natural direct effect | 1.030 (0.972-1.094) | 0.515 |
|  |  | Total effect | 1.038 (0.979-1.103) | 0.361 |
| Alcohol | Ischemic heart disease | Proportion mediated | 0.083 | 0.174 |
|  |  | Pure natural indirect effect | **1.005 (1.001-1.010)** | 0.044 |
|  |  | Natural direct effect | 1.057 (0.991-1.126) | 0.186 |
|  |  | Total effect | 1.062 (0.996-1.132) | 0.163 |
| Alcohol | Heart failure | Proportion mediated | 0.066 | 0.092 |
|  |  | Pure natural indirect effect | **1.005 (1.001-1.010)** | 0.044 |
|  |  | Natural direct effect | 1.073 (1.007-1.144) | 0.082 |
|  |  | Total effect | 1.078 (1.011-1.148) | 0.067 |
| Alcohol | Cerebrovascular disease | Proportion mediated | 0.052 | 0.061 |
|  |  | Pure natural indirect effect | **1.004 (1.001-1.008)** | 0.044 |
|  |  | Natural direct effect | 1.074 (1.015-1.134) | 0.047 |
|  |  | Total effect | 1.078 (1.019-1.139) | 0.036 |
| Alcohol | Peripheral vascular disease | Proportion mediated | 0.046 | 0.044 |
|  |  | Pure natural indirect effect | **1.008 (1.002-1.016)** | **0.044** |
|  |  | Natural direct effect | 1.198 (1.090-1.313) | <0.001 |
|  |  | Total effect | 1.208 (1.099-1.324) | <0.001 |
| Alcohol | Hypertension | Proportion mediated | 0.074 | 0.176 |
|  |  | Pure natural indirect effect | **1.003 (1.001-1.006)** | 0.044 |
|  |  | Natural direct effect | 1.039 (0.994-1.085) | 0.194 |
|  |  | Total effect | 1.042 (0.997-1.088) | 0.163 |
| Alcohol | Obesity | Proportion mediated | 0.033 | 0.044 |
|  |  | Pure natural indirect effect | **1.004 (1.001-1.008)** | 0.044 |
|  |  | Natural direct effect | 1.130 (1.069-1.195) | <0.001 |
|  |  | Total effect | 1.135 (1.072-1.201) | <0.001 |
| Alcohol | Type 2 diabetes | Proportion mediated | 0.087 | 0.512 |
|  |  | Pure natural indirect effect | **1.004 (1.001-1.009)** | 0.047 |
|  |  | Natural direct effect | 1.041 (0.957-1.134) | 0.537 |
|  |  | Total effect | 1.045 (0.961-1.139) | 0.505 |
| Alcohol | Type 1 diabetes | Proportion mediated | 0.022 | 0.222 |
|  |  | Pure natural indirect effect | 1.004 (0.999-1.011) | 0.194 |
|  |  | Natural direct effect | 1.202 (1.028-1.419) | 0.067 |
|  |  | Total effect | 1.207 (1.031-1.424) | 0.058 |
| Alcohol | Rheumatoid arthritis | Proportion mediated | 0.032 | 0.056 |
|  |  | Pure natural indirect effect | 1.004 (1.001-1.009) | 0.054 |
|  |  | Natural direct effect | 1.132 (1.041-1.231) | 0.013 |
|  |  | Total effect | 1.136 (1.045-1.234) | 0.013 |
| Alcohol | Psoriasis | Proportion mediated | 0.014 | 0.725 |
|  |  | Pure natural indirect effect | 1.001 (0.999-1.004) | 0.608 |
|  |  | Natural direct effect | 1.058 (0.972-1.153) | 0.350 |
|  |  | Total effect | 1.059 (0.973-1.153) | 0.345 |
| Alcohol | Inflammatory bowel disease | Proportion mediated | 0.174 | 0.943 |
|  |  | Pure natural indirect effect | 1.002 (0.999-1.007) | 0.402 |
|  |  | Natural direct effect | 1.010 (0.886-1.158) | 0.940 |
|  |  | Total effect | 1.013 (0.888-1.159) | 0.929 |
| Alcohol | Migraine | Proportion mediated | -0.002 | 0.929 |
|  |  | Pure natural indirect effect | 1.000 (0.998-1.001) | 0.929 |
|  |  | Natural direct effect | 1.071 (1.022-1.122) | 0.027 |
|  |  | Total effect | 1.070 (1.022-1.122) | 0.027 |
| Alcohol | Epilepsy | Proportion mediated | 0.102 | 0.529 |
|  |  | Pure natural indirect effect | 1.006 (1.001-1.014) | 0.054 |
|  |  | Natural direct effect | 1.056 (0.930-1.198) | 0.566 |
|  |  | Total effect | 1.062 (0.936-1.208) | 0.520 |
| Alcohol | Dementia | Proportion mediated | 0.105 | 0.569 |
|  |  | Pure natural indirect effect | 1.005 (1.001-1.012) | 0.063 |
|  |  | Natural direct effect | 1.046 (0.937-1.166) | 0.594 |
|  |  | Total effect | 1.051 (0.941-1.173) | 0.567 |
| Alcohol | Parkinson | Proportion mediated | 0.041 | 0.998 |
|  |  | Pure natural indirect effect | 1.000 (0.997-1.003) | 0.943 |
|  |  | Natural direct effect | 1.005 (0.916-1.107) | 0.954 |
|  |  | Total effect | 1.005 (0.916-1.107) | 0.953 |
| Alcohol | Sleep disorders | Proportion mediated | 0.203 | 0.350 |
|  |  | Pure natural indirect effect | **1.008 (1.002-1.015)** | 0.044 |
|  |  | Natural direct effect | 1.032 (0.974-1.096) | 0.473 |
|  |  | Total effect | 1.041 (0.982-1.105) | 0.345 |

**Note:** Table S3 present the total effect, pure natural indirect, and natural direct effects, and the proportion mediated (i.e., ratio of the logit for the indirect effect to the logit for the total effect) by each mediator for each somatic health outcome. Associations are expressed as odds ratios together with their 95% CIs and the proportion mediated as the % of the total effect that is mediated. BMI was mean centered and treated as a continuous variable. Bolded estimates highlight the significant pure natural indirect effects. **Abbreviations:** BMI, body mass index. OR, odds ratio. CI, confidence interval. PRS, polygenic risk score.

# Table S4. Associations of ADHD-PRS with somatic outcomes stratified by sex (N=10,645)

| **Disease area/outcome** | **Males** | | | **Females** | | |  |
| --- | --- | --- | --- | --- | --- | --- | --- |
|  | **N cases males** | **OR (95%CI)** | ***p*_adj_** | **N cases females** | **OR (95%CI)** | ***p*_adj_** | ***P*_interaction_** |
| ***Cardio-metabolic*** |  |  |  |  |  |  |  |
| Ischemic heart disease | 720 | 1.06 (0.98-1.16) | 0.306 | 486 | 1.06 (0.96-1.17) | 0.401 | 0.992 |
| Heart failure | 620 | 1.08 (0.99-1.18) | 0.290 | 554 | 1.08 (0.98-1.18) | 0.245 | 0.992 |
| Cerebrovascular disease | 1098 | 1.08 (1.01-1.17) | 0.200 | 588 | 1.07 (0.98-1.17) | 0.271 | 0.992 |
| Peripheral vascular disease | 300 | 1.14 (1.01-1.29) | 0.200 | 223 | 1.29 (1.12-1.49) | 0.007 | 0.855 |
| Hypertension | 1251 | 1.04 (0.97-1.12) | 0.349 | 1479 | 1.04 (0.98-1.11) | 0.400 | 0.992 |
| Obesity | 765 | **1.14 (1.06-1.24)** | 0.016 | 773 | **1.13 (1.04-1.23)** | 0.026 | 0.992 |
| Type 2 Diabetes | 342 | 1.10 (0.98-1.22) | 0.290 | 212 | 0.98 (0.84-1.14) | 0.795 | 0.855 |
| ***Autoimmune/inflammatory disease*** | |  |  |  |  |  |  |
| Type 1 Diabetes | 109 | 1.17 (0.97-1.41) | 0.290 | 54 | 1.31 (0.96-1.79) | 0.235 | 0.992 |
| Rheumatoid arthritis | 178 | 1.11 (0.96-1.28) | 0.306 | 416 | 1.15 (1.04-1.27) | 0.027 | 0.992 |
| Psoriasis | 293 | 0.93 (0.82-1.05) | 0.349 | 312 | 1.19 (1.06-1.34) | 0.026 | 0.136 |
| Inflammatory bowel disease | 113 | 1.07 (0.87-1.31) | 0.620 | 130 | 0.97 (0.81-1.15) | 0.750 | 0.992 |
| ***Neurology*** |  |  |  |  |  |  |  |
| Migraine | 665 | 1.06 (0.98-1.15) | 0.306 | 1542 | 1.08 (1.01-1.14) | 0.049 | 0.992 |
| Epilepsy | 124 | 1.02 (0.87-1.20) | 0.781 | 107 | 1.09 (0.89-1.35) | 0.577 | 0.992 |
| Dementia | 184 | 1.04 (0.89-1.22) | 0.669 | 190 | 1.06 (0.90-1.24) | 0.577 | 0.992 |
| Parkinson disease | 202 | 0.96 (0.83-1.10) | 0.620 | 271 | 1.04 (0.90-1.19) | 0.699 | 0.992 |
| Sleep disorder | 501 | 1.06 (0.96-1.16) | 0.354 | 771 | 1.03 (0.95-1.11) | 0.577 | 0.992 |

**Note**. *P*_adj_, false discovery rate adjusted *p*-values. *P*_interaction,_ *p*-value for the interaction term with sex. Bolded estimates display significant false discovery rate adjusted p-values

**Abbreviations:** OR, odds ratio. CI, confidence interval. PRS.

# Fig. S1: Associations of ADHD-PRS and somatic health outcomes evaluated by register-data and self-reported compared to the combined estimates, expressed by standard deviation of the PRS


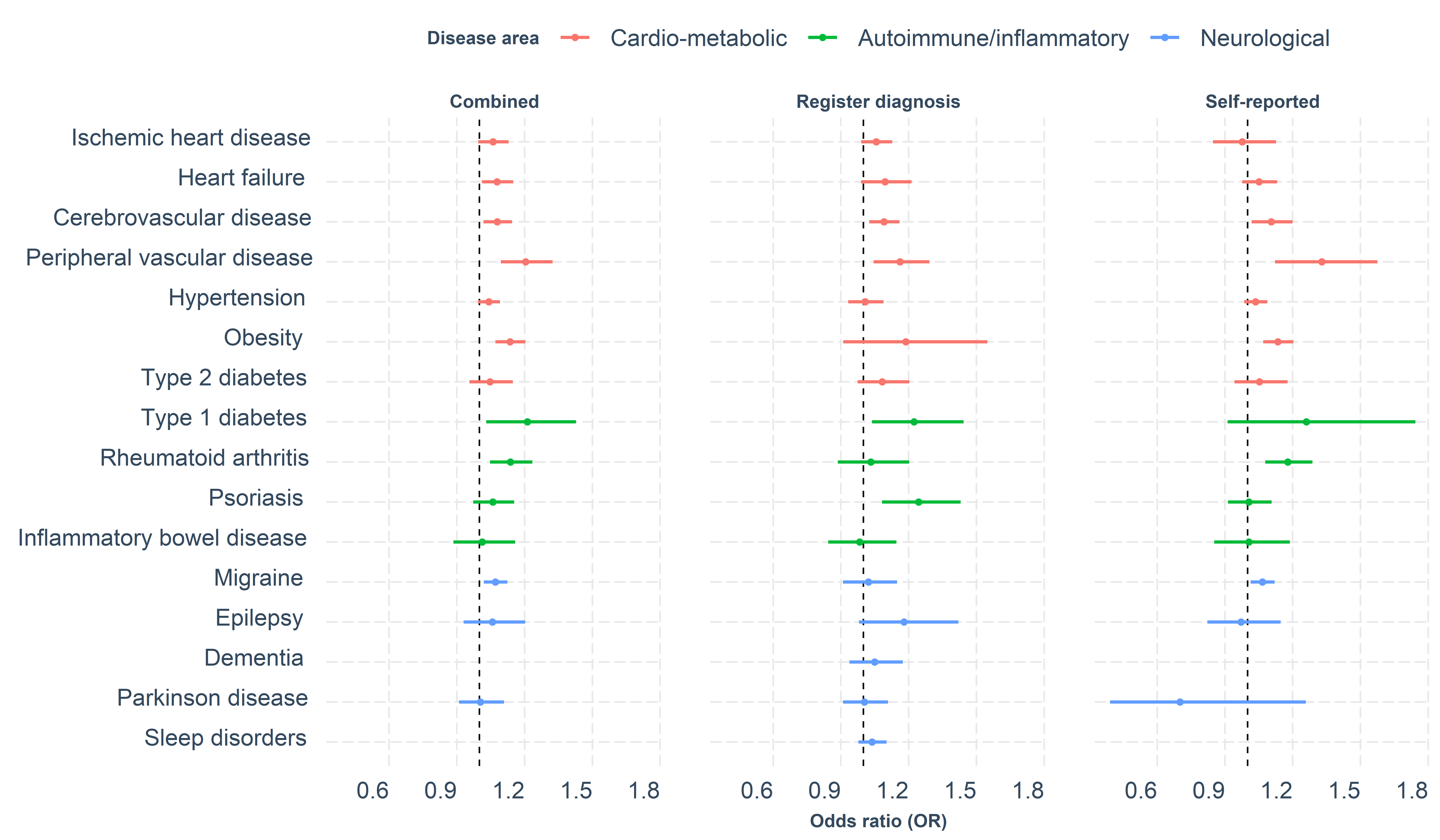


**Note**: Associations are expressed as Odds Ratios (OR) with 95% confidence intervals.

# Fig. S2: NagelKerke pseudo-R^2^ for the associations of ADHD-PRS and somatic health outcomes evaluated by register-data and self-reported compared to the combined estimates, expressed by standard deviation of the PRS


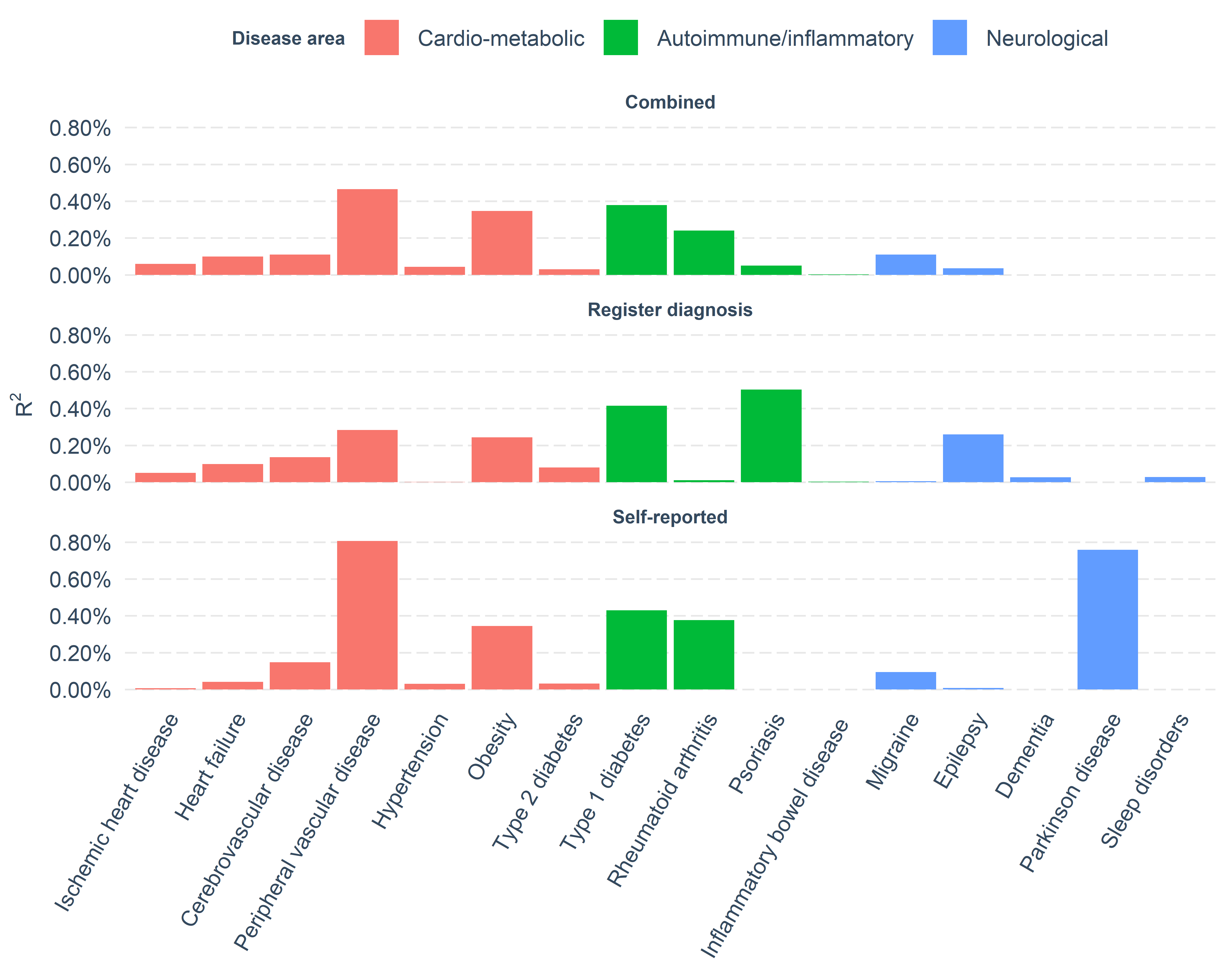


# Fig. S3: Directed acyclic graph illustrating the mediation models


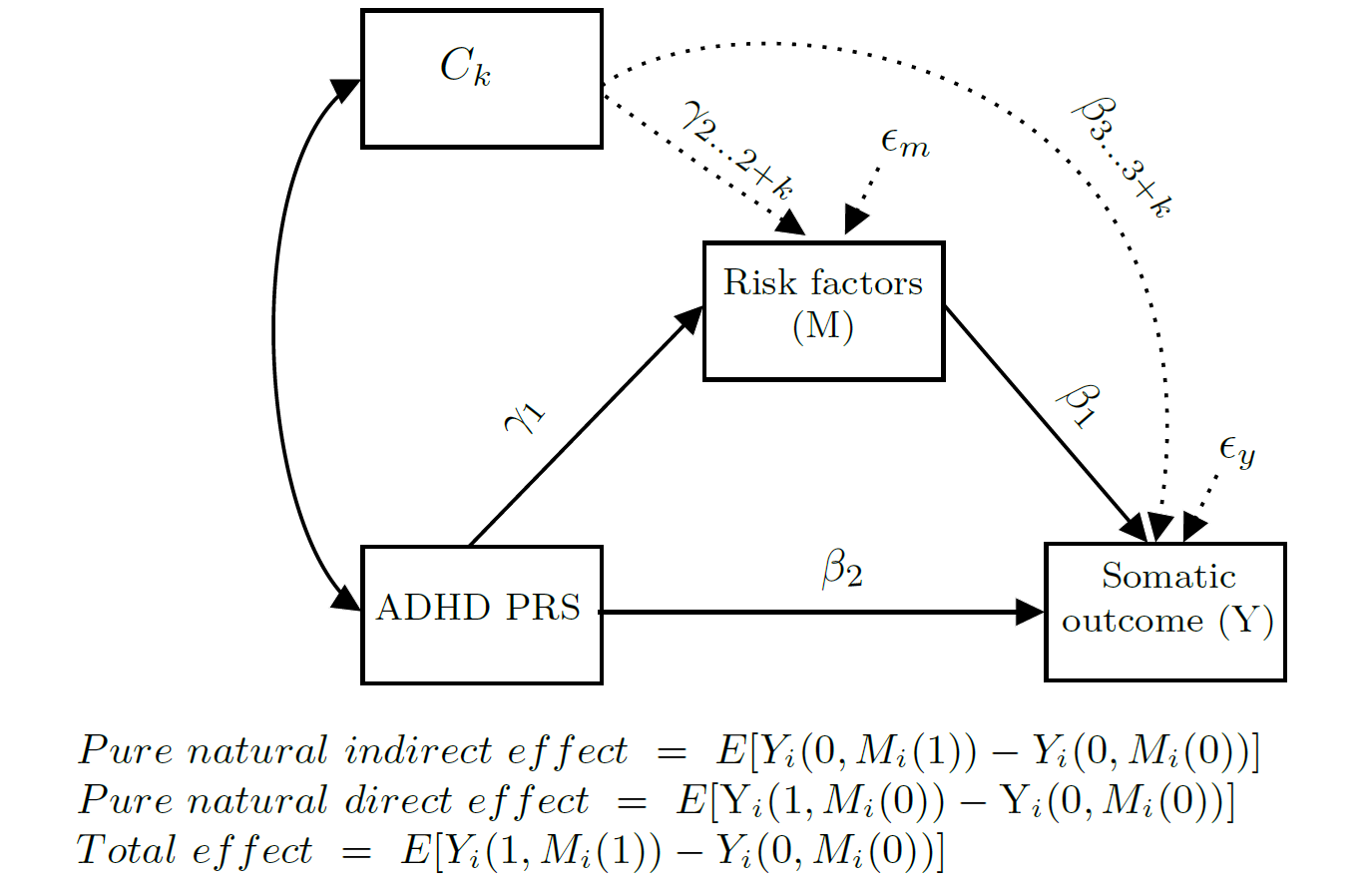


**Note**: The diagram displays the mediation model for $risk factor_{j}$ mediating the relationship between ADHD PRS and somatic $outcome_{i}$ after adjusting for our set of covariates ($C_{k}$)
